# Mechanisms of exercise intolerance in heart failure with preserved ejection fraction (HFpEF)

**DOI:** 10.1007/s10741-025-10504-3

**Published:** 2025-03-13

**Authors:** Brandon Pecchia, Roy Samuel, Vacha Shah, Emily Newman, Gregory T. Gibson

**Affiliations:** 1 Department of Medicine, Sidney Kimmel Medical College at Thomas Jefferson University, Philadelphia, PA USA; 2 Sidney Kimmel Medical College at Thomas Jefferson University, Philadelphia, PA USA; 3 Division of Cardiology, Department of Medicine, Sidney Kimmel Medical College at Thomas Jefferson University, Philadelphia, PA 19107, US, Philadelphia, PA 19107 USA

**Keywords:** Heart failure, HFpEF, Exercise, Review

## Abstract

Exercise intolerance is a well-established symptom of heart failure with preserved ejection fraction (HFpEF) and is associated with impaired quality of life and worse clinical outcomes. Historically attributed to diastolic dysfunction of the left ventricle, exercise intolerance in HFpEF is now known to result not only from diastolic dysfunction, but also from impairments in left ventricular systolic function, left atrial pathology, right ventricular dysfunction, and valvular disease. Disorders of heart rate and rhythm such as chronotropic incompetence and atrial fibrillation have also been implicated in exercise intolerance in this population. Pathologic changes to extra-cardiac organ systems including the respiratory, vascular, hormonal, and skeletal muscle systems are also thought to play a role in exercise impairment. Finally, comorbidities such as obesity, inflammation, and anemia are common and likely contributory in many cases. The role of each of these factors is discussed in this review of exercise intolerance in patients with HFpEF.

## Introduction

Heart failure is currently defined as a clinical syndrome with current or prior signs or symptoms caused by a structural or functional cardiac abnormality and corroborated by objective evidence of congestion. Heart failure can be further classified by ejection fraction (EF). Patients with an EF ≥ 50% are defined as having heart failure with preserved ejection fraction (HFpEF) [1]. Subclassification on the basis of EF exists largely to facilitate the distinction between HFpEF and heart failure with reduced ejection fraction (HFrEF, defined as EF ≤ 40%), which may indicate heart failure syndromes resulting from different underlying disease processes. HFpEF comprises 50% of all heart failure syndromes, is associated with a significant burden of comorbidities, and appears to be increasing in prevalence [2–4]. Relating to its high burden of comorbidities, HFpEF syndromes are known to be quite heterogeneous, with multiple distinct phenotypes having been proposed [5–7]. Beyond diastolic dysfunction of the left ventricle, pathology involving the vascular, pulmonary, hepatic, renal, and skeletal muscle systems has been identified [7, 8].

Despite the degree of heterogeneity amongst those with HFpEF, exercise intolerance is a shared feature and a hallmark of this clinical syndrome [9]. Previous work has indicated that patients with HFpEF may experience a reduction in peak oxygen uptake (VO_2_) of approximately 35% as assessed by cardiopulmonary exercise testing (CPET) when compared to healthy age- and sex-matched controls [10]. As a result, many of these patients have a measured peak VO_2_ in the range of 9–15 mL/kg/min [11, 12], which is a degree of exercise impairment that is similar to those with advanced HFrEF [13, 14].

The severity of exercise impairment in patients with HFpEF has been associated with poor outcomes. For example, reductions in distance on a 6-min walk distance (6MWD), have been demonstrated, with lower distances shown to be an independent predictor for death or HF-related hospitalizations [15]. Reductions in peak VO_2_ have also been shown to be associated with worse clinical outcomes in patients with HFpEF [16, 17].

Exercise intolerance in patients with HFpEF is often significant enough to adversely impact the ability to perform activities of daily living and consequently is associated with reduced quality of life [9, 13, 18]. However, there is evidence to suggest that those with HFpEF may benefit from exercise training and treatment of associated comorbidities [19]. In this review, we will summarize the proposed underlying mechanisms of exercise intolerance in patients with HFpEF.

## Cardiovascular response to exercise in healthy individuals

In order to appreciate the underlying pathophysiology of exercise limitations in patients with HFpEF, it is instructive to briefly review the normal physiologic changes which occur within the cardiovascular system during exercise. Exercise capacity is often quantified as a measure of oxygen uptake (VO_2_), which is defined by the Fick principle as the product of cardiac output (CO) and the arterial and mixed venous oxygen content difference [20]. Reduced VO_2_ at peak exercise is a hallmark of exercise intolerance in patients with HFpEF [10, 21, 22]. Facilitation of increased oxygen delivery and extraction by the periphery during exercise relies on several mechanisms. First is an increase in cardiac output, which is a function of the product of stroke volume (SV) and heart rate (HR). Augmentation of SV occurs in part due to increases in ventricular end-diastolic volume (EDV), which results in increased end-diastolic pressure, which is a major, but not sole determinant of ventricular preload. In turn, increased preload leads to further stretch of cardiac myocytes and thus increased force of contraction by the Frank-Starling mechanism. It is important to note that this increased force of contraction is a result of the optimization of sarcomere length and muscle fiber tension, and not due to increased inotropy, which is a separate process mediated by neurohormonal mechanisms. During exercise, increased right ventricular (RV) preload occurs in response to augmented venous return. This is partially modulated by changes in splanchnic blood volume [23] and further modulated by the effect of contracting skeletal muscle which enhances venous return (a mechanism known as the “skeletal muscle pump”) [24]. Increased right ventricular SV leads to augmented filling of the left heart and a rise in left ventricular EDV, which consequently results in increased left ventricular SV. In healthy subjects, the ventricular diastolic function is adequate to accommodate increases in EDV without the excessive rise in EDP that is associated with exercise intolerance in patients with HFpEF. In addition to increased contractility due to the Frank-Starling mechanism, a rise in sympathetic outflow further contributes to increased contractility via activation of beta receptors. Increased HR, the other major determinant of cardiac output during exercise, also occurs as a response to changes in autonomic tone [25]. In fact, augmentation of cardiac output during higher levels of exercise is thought to be primarily due to increased heart rate rather than augmented SV [26]. Collectively, oxygen uptake has been demonstrated to increase nearly eightfold during maximal exercise, with cardiac index increasing approximately threefold as a result of augmentation of HR (2.5-fold), left ventricular SV (1.5-fold), and peripheral oxygen utilization (2.5-fold) in healthy men [26].

## Left heart dysfunction

### Left ventricle

Traditionally, the primary pathophysiologic mechanism of elevated filling pressures and exercise intolerance in patients with HFpEF has been attributed to diastolic dysfunction of the left ventricle (LV). Diastolic dysfunction of the LV is a result of impaired relaxation, which is an energetically active process. The mechanisms accounting for impaired relaxation are thought to include abnormal calcium regulation [27], hypertrophy of the myocardium, and changes to the extramyocardial collagen matrix [28, 29]. Diastolic dysfunction of the left ventricle results in an upward shift in the pressure–volume relationship, such that higher left ventricular end-diastolic pressures (LVEDP) occur at lower end-diastolic volumes (EDV). While EDV does not appear to be significantly impaired in patients with HFpEF during exercise [11, 30, 31], disproportionately increased LVEDP has been demonstrated [32, 33]. The observed increases in LVEDP have been shown to occur early in exercise and resolve quickly upon cessation [12, 34, 35].

Despite a normal ejection fraction at rest, there is ample evidence that LV systolic function is also impaired in this population. Prior work has repeatedly demonstrated reductions in ejection fraction and global longitudinal strain with exercise [36–39] in patients with HFpEF. Lack of contractile reserve may contribute to decreased cardiac output in this population, with a corresponding decrease in peak VO_2_. LV systolic dysfunction may be mediated by multiple factors, many of which are vascular in nature. These include microvascular dysfunction and reduced peripheral arterial compliance (particularly in the aorta) which results in impaired ventricular-vascular coupling [40–43]. Mechanisms of vascular dysfunction in patients with HFpEF will be further discussed elsewhere.

### Left atrium

In addition to the abnormalities in the left ventricle, structural and functional changes in the left atrium have also been implicated in the development of HFpEF and in its associated exercise intolerance [44–47]. Normally, the left atrium functions through multiple phases of the cardiac cycle as a reservoir, conduit, and pump [48]. Adequate left atrial compliance and pumping function during atrial systole are necessary in order to maintain low pressures in the pulmonary venous system. Changes to left atrial structure, notably left atrial enlargement, have been associated with fibrotic changes and reductions in function, including a loss of compliance [49, 50]. Left atrial size is known to proportionally increase as a result of elevated left atrial pressures, with progression related to the chronicity and intensity of these pressure changes [48, 51, 52]. Indeed, left atrial enlargement has been shown to be an independent predictor of poor exercise capacity [53]. Further, impairment in left atrial function has been demonstrated in patients with HFpEF both at rest and during exercise [44, 46, 47, 54]. Finally, adverse left atrial structural changes are associated with atrial fibrillation, which itself contributes to exercise intolerance in patients with HFpEF [55].

## Pulmonary hypertension and right ventricular function

Pulmonary hypertension resulting from left heart disease has been estimated to be present in approximately 80% of patients with HFpEF and is associated with reduced survival [56–58]. There are a variety of contributing factors which may be present in a patient with HFpEF, including LV dysfunction (both systolic and diastolic), reduced left atrial compliance, and valvular disease, amongst others. In many instances, elevations in LVEDP result in elevated left atrial pressure, pulmonary venous hypertension, and subsequently pulmonary arterial hypertension. On a chronic basis, elevated pulmonary arterial pressures can lead to pulmonary vascular remodeling and increased pulmonary vascular resistance (PVR). While pulmonary hypertension is present in a resting state in many patients with HFpEF, those with normal or near pulmonary arterial (PA) pressures at rest may experience a rapid increase in PA pressures shortly after the onset of exercise. This has been shown in previous work by Borlaug et al., who demonstrated rapid increases in pulmonary capillary wedge pressure with increased preload and with exercise [34]. The relationship between cardiac output (CO) and mean PA pressure (mPA) has also been shown to be altered in this group, with a greater increase in mPA relative to CO (increased mPA/CO slope) [59]. These findings reflect the effects of impaired left ventricular diastolic function and reduced left atrial compliance, which are exacerbated by exercise. Consequently, elevated pulmonary venous and pulmonary capillary pressures can lead to pulmonary edema, hypoxia, and metabolic acidosis, which limit exercise capacity. Additionally, these effects can lead to uncoupling of the normal RV-PA relationship [59], and worsening RV function.

## Heart rate and rhythm

As previously discussed, elevation of heart rate is a key component of the normal response to exercise in healthy individuals and is the primary contributor to cardiac output augmentation during later stages of exercise [26]. The inability to appropriately increase HR with exercise, or chronotropic incompetence, has long been observed in patients with HFpEF with an estimated prevalence of approximately 30–50% [60]. This effect is significant, with one study demonstrating a mean reduction in peak heart rate of nearly 25%, or 39 beats/min, compared to controls of similar age group [60]. Chronotropic incompetence may be a result of multiple factors, including the use of beta blockers, which the blunt the heart rate response to exercise. Recent work has demonstrated that withdrawal of beta blockers in patients with HFpEF leads to improvement in exercise capacity [61].

### Atrial fibrillation

Atrial Fibrillation is a well-established risk factor for HFpEF, with an estimated prevalence ranging from 39 to 65% in this population [62–65]. Independently, atrial fibrillation is associated with a reduction in peak VO_2_ of approximately 1–3 mL/kg/min during exercise, a finding that has been shown to be at least partially reversible following restoration of sinus rhythm [66–71]. In patients with HFpEF and permanent atrial fibrillation, increased congestion and lower cardiac output have been noted during exercise testing with invasive hemodynamics [45]. At least one study also observed signs of ventilatory inefficiency as evidenced by an increased minute ventilation (VE)/VCO_2_ slope, which itself is a poor prognostic marker in patients with heart failure [68]. Finally, patients with HFpEF and atrial fibrillation may also be subject to chronotropic incompetence, at least in part due to the effect of AV nodal blockers.

## Systemic and pulmonary vascular effects on exercise intolerance in HFpEF

Changes in arterial function play a role in exercise intolerance in patients with HFpEF. Endothelial microvascular dysfunction, when measured with digital artery tonometry, was more apparent in patients with HFpEF than in controls [72]. This dysfunction correlated with reduced exercise capacity assessed by lower peak oxygen uptake. Additionally, invasive measures of arterial stiffness in patients with HFpEF in a study by Zern et al. demonstrated that at rest, HFpEF patients had poorer measures of arterial stiffness than controls. Exercise further exacerbated the differences in arterial stiffness, and there was a direct relationship between arterial stiffness and an abnormal increase in PCWP during exercise that corresponded with lower oxygen uptake [72].

Mechanistically, a defective microvascular reserve could result in impaired or reduced oxygen transport via diffusion to active muscles, reducing exercise tolerance [73]. Moreover, many of the comorbidities associated with HFpEF, such as obesity, diabetes mellitus, and hypertension further contribute to endothelial dysfunction and systemic inflammation [74]. This systemic endothelial inflammation causes the release of reactive oxygen species by coronary microvascular endothelial cells, which, through subsequent signaling pathways and reduced nitric oxide (NO) production, results in increased cardiomyocyte stiffness and hypertrophy, as well as interstitial collagen deposition through myofibroblast formation [75]. Altogether, this aberrant cascade induces diastolic LV dysfunction [76].

HFpEF is also accompanied by pulmonary vascular remodeling [77]. Pulmonary venous endothelial dysfunction can arise due to mechanical distension of the pulmonary veins that results in the activation of growth factors promoting intimal fibrosis. Additionally, pulmonary venous hypertension can directly cause venous endothelial damage [78]. In a comparison of patients with HFpEF with and without pulmonary vascular disease, exercise uncovered a distinct pathophysiology in the former group: there was impaired blood flow through the lungs, subsequent right heart congestion, and ventricular interdependence that decreased stroke volume and prevented an increase in cardiac output during exercise [79]. It was also observed that pulmonary vascular disease was associated with worse systemic arterial disease and with a higher mean vascular resistance [79]. Systemic vascular stiffening in HFpEF described above is associated with exercise-induced pulmonary hypertension that is partially reversible with the administration of NO therapies [79]. Thus, endothelial dysfunction and deficiencies in NO production play crucial roles in both the systemic and pulmonary vasculature in HFpEF [75].

## Pulmonary/respiratory system

HFpEF is associated with a number of derangements in the pulmonary system, including pulmonary vascular congestion, pulmonary hypertension, impaired alveolar gas exchange, and increased ventilation/perfusion mismatch. During exercise, impairments in left ventricular diastolic function lead to elevations in left atrial pressure. Increased left atrial pressure, in turn, causes increases in pulmonary venous and capillary pressures, resulting in pulmonary hypertension. In patients with long-standing resting elevation of the left heart filling pressures, structural changes in small pulmonary arteries can occur, including medial hypertrophy and intimal thickening. These structural changes result in decreased pulmonary vascular compliance. Subsequent reductions in pulmonary vascular compliance further the propensity for the development of pulmonary hypertension with exercise, as the pulmonary circulation is unable to accommodate the increased transpulmonary flow that occurs with augmented cardiac output. Further, such structural abnormalities can precipitate a phenomenon known as capillary stress failure, where elevated pulmonary pressures disrupt endothelial function, increasing the permeability of fluids, proteins, and cells to the alveolar space. This infiltration decreases the available surface area for gas exchange and results in impaired diffusing capacity, which can lead to hypoxia [80].

Exercise intolerance in HFpEF can also be explained in part by respiratory muscle dysfunction. Respiratory muscle weakness was recently identified in HFpEF, leading to inefficient ventilation and thus dyspnea. This was also found to be associated with increased all-cause mortality (likely through increased respiratory complications and CV events) [81]. Indeed, inspiratory muscle weakness is associated with exercise intolerance in patients with HFpEF [82], and exercise training [83], including inspiratory muscle training, has been shown to improve peak VO_2_, 6-min walk distance, and quality of life [84, 85].

## Musculoskeletal abnormalities

The effects of heart failure on the musculoskeletal system are multifactorial, with various explanations for the downstream effects and complex interplay amongst other physiologic processes. The general concept is that there are both “central” components (i.e., cardiac output) and “peripheral” components (changes in skeletal muscle) that contribute to exercise intolerance. While the basic contribution is often explained by LV dysfunction leading to decreased skeletal muscle perfusion and decreased O_2_ utilization, this is further complicated by competing respiratory and limb skeletal muscle, and an increased catabolic state that contributes to skeletal muscle myopathy [86].

The metabolic and structural changes which occur in skeletal muscle in patients with HFpEF have been well-described [87, 88]. In addition to reductions in lean body mass, lower levels of Type 1 (“slow-twitch”) oxidative muscle fibers have been observed [89]. Type 1 fibers rely primarily on oxidative metabolism via mitochondria to generate adenosine triphosphate (ATP), and mitochondrial dysfunction appears to play a significant role in the functional impairment of skeletal muscle [90]. Mitochondrial dysfunction results not only in a diminished capacity to produce ATP via oxidative phosphorylation but also leads to dysregulation of calcium homeostasis and increased oxidative stress. Additionally, such metabolic derangements result in diminished nitric oxide bioavailability, leading to endothelial and cardiomyocyte dysfunction [91]. Summarily, such pathologic alterations in skeletal muscle structure and function are thought to contribute to a reduction in peripheral oxygen extraction and reduced peak VO2 in patients with HFpEF [87, 92].

Beyond primary changes in skeletal muscle, peripheral mechanisms have also been implicated. These include a phenomenon known as the ergoreflex: a term coined to describe the cardiorespiratory reflex that acts as a feedback system during physical activity. This is further broken down into an earlier component, the mechanoreflex: brought about by mechanical stretch of the muscles and tendons, followed by the metaboreflex: formed by the buildup of metabolites such as lactic acid, prostaglandins, bradykinin, and hydrogen ions within the muscle. Both reflexes are believed to increase ventilation, vascular resistance, and cardiac output, primarily elicited by increased sympathetic outflow, in order to maintain an adequate perfusion to muscles during exercise [93].

A dominating concept in heart failure is the “muscle hypothesis,” a vicious cycle where skeletal myopathy increases the ergoreflex sensitivity, thus leading to increased exertional dyspnea and sympathetic activation. When chronically stimulated, the increased sympathetic drive (primarily via vasoconstriction) can negatively alter the structure and function of the myocardium, by contributing to increased afterload, putting further strain on the left ventricle, decreasing skeletal muscle perfusion, and causing even more myopathy [93–95]. Symptoms intensify, patients avoid activity, which further promotes the onset of dyspnea and fatigue, leading to further physical inactivity and exercise intolerance. This was shown to be at least partially reversible with exercise training [96]. As previously described, part of the perceived dyspnea in HFpEF is also due to resultant respiratory muscle weakness, which further adds to the cycle [81].

More recent data have shown that the diastolic impairment seen in HFpEF also disrupts the normal metaboreflex effects, preventing the increase in stroke volume and cardiac output typically elicited by the postexercise muscle ischemia method, a reproducible protocol that has been shown to provoke the metaboreflex in isolation [97]. In contrast, individuals with diastolic dysfunction were not able to mount an augmentation in EDV, SV, or CO, and instead relied on an exaggerated vasoconstriction to attain the goal MAP that the exercise demanded. This further corroborates the concept that in HFpEF, diastolic dysfunction diminishes preload reserve, and attaining a target blood pressure to maintain exercise levels shifts from a CO-mediated response to a systemic vascular resistance (SVR)-mediated response [97].

As noted, exercise training in patients with HFpEF improves exercise tolerance, primarily through the peripheral components, with minimal effect on cardiac output [98]. Data suggest that exercise training may reduce that oversensitization of the ergoreflex, likely mediated in part by restoring autonomic balance [93]. Part of the benefit of exercise training is to “blunt” the ergoreflex sensitivity, thereby decreasing the fatigue and perceived dyspnea, and the whole cascading spiral of secondary effects described above.

## Neurohormonal effects on exercise intolerance in HFpEF

Regardless of the underlying etiology, HFpEF is associated with alterations in neuroendocrine function. These changes include increased activation of the renin–angiotensin–aldosterone system (RAAS), which occurs concomitantly with enhanced sympathetic activity and decreased vagal output [99]. Briefly, with a fall in cardiac output, the sympathetic nervous system and RAAS are activated, resulting in increased levels of circulating norepinephrine and angiotensin II. These neurohormones promote peripheral vasoconstriction as well as the secretion of aldosterone. While these neurohormones are initially beneficial, they become deleterious when chronically sustained [100].

Pharmacological therapies to address neurohormonal modulation in patients with HFpEF are limited. While beta blockers should limit sympathetic nervous system activation, these benefits may be outweighed by their negative chronotropic effect, especially since the inability to increase heart rate in response to increased demand is a major player in exercise intolerance in HFpEF [61]. As of now, their use is primarily in patients who additionally have a history of myocardial infarction or for rate control in atrial fibrillation [101].

More recently, sodium-glucose cotransporter-2 inhibitors (SGLT2i) have been demonstrated to improve exercise capacity in patients with HFpEF [102]. The underlying mechanisms for this benefit remain under investigation, but they appear to be multifactorial. For example, in a study of patients treated with dapagliflozin, there were noted to be reductions in body weight, plasma volume, and PCWP both at rest and during exercise [103]. An increase in pulmonary artery compliance and improvement in right ventricular function was identified, which appear to be related reductions in PCWP [104]. Lastly, this study cohort was found to have an improvement in arterial compliance and venous capacitance during exercise, suggesting that SGLT2i may improve exercise capacity through beneficial effects on vascular function [105].

## Other contributory mechanisms of exercise intolerance

Beyond key changes in the structure and function of the cardiovascular, pulmonary, skeletal, and neuroendocrine systems, numerous other abnormalities have also been implicated in patients with HFpEF and exercise intolerance. Several of these topics will be described below, in addition to a discussion of some emerging theories of contributing mechanisms.

### Valvular disease

Valvular disease, while not necessarily a primary component of HFpEF, can be coexistent in this patient population, and contribute to impaired exercise capacity. The degree to which valvular lesions may impact exercise in patients with HFpEF is dependent upon the location and severity of the valvular abnormality. Two lesions that are commonly observed in patients with HFpEF include aortic stenosis and mitral regurgitation. In patients with calcific aortic stenosis, recent work has demonstrated similar reductions in peak VO_2_ and cardiac output compared to those with HFpEF [106]. Another study of patients with HFpEF with and without mitral regurgitation (MR) found that those with MR had reduced cardiac output with exercise, impaired pulmonary vasodilation, and blunted right ventricular reserve [107]. These patients were also noted to have more severe left atrial enlargement and decreased compliance, suggesting that chronic MR can worsen left atrial function.

### Anemia

Anemia is highly prevalent in patients with HFpEF, with an estimated prevalence of over 40% [108, 109]. Such patients often present with co-morbid anemia due to various etiologies, including chronic blood loss due to anticoagulation agents, cardiorenal syndrome, chronic inflammation, poor nutritional status, and iron deficiency [109]. Anticoagulant use in HFpEF reduces thromboembolic events caused by endothelial dysfunction, arrhythmias, and atrial blood stasis [110]. All of these agents increase bleeding risk which can cause anemia by loss of red blood cells. In patients with cardiorenal syndrome, renal insufficiency as a result of chronic venous hypertension leads to decreased production of erythropoietin (EPO) and subsequently decreased hematopoiesis [111]. Anemia may also be precipitated by a state of chronic inflammation in patients with heart failure [112].

In addition to the inherently decreased oxygen-carrying capacity and oxygen delivery seen in anemia, which results in decreased peak VO_2_, there also appear to be implications of iron deficiency, even independent of anemia. The majority of studies investigating these effects have been carried out in HFrEF. In patients with iron deficiency and HFrEF, the FAIR-HF and the FERRIC-HF trials, among others, found that intravenous iron therapy significantly improved endurance capacity and symptoms, evidenced by improvement in peak VO_2_, increased in distance on 6-min walk test, and improvement in New York Heart Associated (NYHA) functional class, as well as improved quality of life (QOL) [113, 114]. FERRIC-HF showed more robust findings in anemic patients, though FAIR-HF identified these changes in those with iron deficiency in the absence of anemia.

The AFFIRM-AHF trial, as well as a systematic review and meta-analysis, did not show reductions in mortality with iron supplementation, but did find significant reductions in heart failure hospitalizations and overall hospitalizations [115]. Both ESC and ACC/AHA/HFSA guidelines now recommend consideration for intravenous (IV) iron in heart failure to improve functional status and QOL [101, 116]. While it is unclear whether these findings can be generalizable to patients with HFpEF, the FAIR-HFpEF trial is underway to investigate the effects of IV iron on exercise tolerance, symptoms, and quality of life in patients with iron deficiency, with and without anemia [117]. The PREFER-HF trial will evaluate the effect of iron supplementation (both IV and oral) on symptoms and functional class in patients with HFpEF [118].

### Obesity/inactivity

The link between obesity, physical inactivity, and HFpEF has been well-established [119], with estimates of about half of patients with HFpEF being obese, and over 80% being overweight or obese [120, 121]. There are several additional proposed mechanisms for the relationship between obesity and exercise intolerance in patients with HFpEF. A unique phenotype of obese HFpEF has been identified, which is associated with reduced exercise capacity (decreased peak VO_2_) [122, 123], higher filling pressures in both left and right ventricles during exercise [5], as well as worse NYHA class, and even worse quality of life [123].

First, excess adipose that accumulates in muscle tissue is also correlated with muscle weakness and impaired exercise performance with intramuscular fat, as well as the ratio of intramuscular fat and skeletal muscle being independent predictors of peak VO_2_ [22]. There are also greater degrees of LV concentric remodeling in the described “obese phenotype” of HFpEF [5]. Additionally, the severity of RV dysfunction has also been noted to be more profound in the obese phenotype than the non-obese phenotype [124]. Further, there is evidence of more profound exercise-induced increase in PA pressures and insufficient pulmonary vasodilation in the “obese” HFpEF phenotype compared to the non-obese phenotype [5].

There is also increased epicardial adipose tissue seen in the obese phenotype, which is associated with greater total heart volume, more pericardial restraint, and greater septal flattening, suggesting greater ventricular interdependence [125]. This may further affect the RV during exercise due to a more confined space to contend with during the expected increased venous return [5]. Among obese patients with HFpEF, there is indeed evidence of those with epicardial adipose tissue being associated with higher filling pressures, greater ventricular interdependence, and decreased peak VO_2_ [126]. Of note, the group with the epicardial adipose tissue did have a significantly higher BMI, but whether the contributions to these above changes are due specifically to the epicardial adipose tissue, or the overall BMI, and thus possibly non-cardiac adipose tissue as well, cannot be fully delineated.

Intriguingly, the use of glucagon-like peptide 1 receptor (GLP-1) agonists, which were originally developed for the treatment of diabetes mellitus and obesity, have been shown to improve exercise capacity in patients with HFpEF [127, 128]. Whether the beneficial effects of GLP-1 agonists on exercise capacity are simply due to weight loss, or a result of other pleiotropic effects requires further investigation. Nonetheless, these findings suggest that treatment targeting the comorbidities present in a particular phenotype may be a more efficacious strategy to improve exercise intolerance than a more generalized approach.

### Postural differences in exercise response

A clinical diagnosis of HFpEF can often be made with a routine cardiovascular evaluation which demonstrates elevated filling pressures and congestion at rest. However, a proportion of patients are known to have normal resting filling pressures, and thus exercise testing is required to make a diagnosis of HFpEF [34, 129]. As discussed earlier, such affected patients typically develop brisk increases in PCWP during exercise, indicating left heart dysfunction, along with reduced exercise capacity.

It is important to recognize that differences in exercise capacity have been observed depending on the modality of exercise being performed, with greater Peak VO_2_ and heart rate response noted when subjects use a treadmill compared to recumbent ergometer [130]. Hemodynamic criteria for HFpEF also vary based on the modality of exercise that is utilized [129]. Specifically, recent studies have demonstrated differences in the hemodynamic response in supine vs. upright exercise testing. For example, Fudim et al. [131] performed both supine and upright ergometer testing and found that only half of those subjects who met the criteria for HFpEF based on the results of supine testing also did so with upright exercise. From a mechanistic perspective, supine exercise with an ergometer requires a leg-lift maneuver in order to maintain a pedaling position, leading to increased venous return and higher atrial pressures than during upright exercise [132–134]. It has been postulated that the increased volume loading associated with supine exercise may not be reflective of the conditions in which typical upright exercise occurs and could result in an overdiagnosis of HFpEF [131]. It should be noted, however, that despite subjects with discordant findings on exercise testing having lower resting filling pressures, rates of atrial fibrillation, and fewer structural abnormalities, they exhibited similar severities of exercise impairment.

### HFpEF phenotypes and sex differences

It has become clear that HFpEF is indeed a widely heterogeneous disease, and the recent emergence of the concept of distinct phenotypes is worth noting. In addition to mounting evidence of an “obese” phenotype, others have also been proposed. For example, Teramoto et al. described multiple HFpEF phenotypes with variable regional propensities, including, “Diabetic obese” (North America), “Hypertensive women” (Latin America), and, “Young ischemic” (Eastern Europe) [6]. Multiple studies have also demonstrated sex differences in the exercise response in patients with HFpEF. Previous work has shown that while men and women with HFpEF may have similar exercise deficits with respect to peak VO_2_, women appear to have greater impairments in peripheral oxygen extraction and both systolic and diastolic reserve despite a lower burden of cardiometabolic disease [135]. Specifically, PCWP has been observed to increase disproportionately in women relative to workload and stroke volume, again suggesting a more prominent reduction in diastolic reserve [136]. Consequently, it stands to reason that those with certain subtypes of HFpEF may experience exercise limitations due to greater functional deficits in a particular system. For example, patients with a “Hypertensive woman” phenotype might be more subject to deleterious changes in vascular function and LV remodeling than those in a “young ischemic” cohort. As such, a “one size fits all” approach to exercise intolerance in patients with HFpEF is perhaps best put aside in favor of more focused, individualistic assessments.

### The role of elevated PCWP during exercise—cause or effect?

A hallmark finding in patients with HFpEF is the rapid increase in PCWP during exercise. As discussed, this has been thought to be primarily due to impairments in LV diastolic function and reductions in left atrial compliance. Such pathologic elevations in left-sided filling pressures and their effects on the pulmonary circulation are widely considered to be a principal cause of exercise intolerance in this population. Consequently, strategies to prevent disproportionate increases in PCWP have been pursued. Both intravenous and inhaled nitrates have previously been demonstrated to reduce PCWP during exercise in patients with HFpEF [137, 138]. However, subsequent trials in which nitrates were administered regularly for an extended period (4–6 weeks) did not show improvement in exercise capacity or activity levels in this cohort [139, 140]. More recently, Sarma et al.[141] performed a study in which participants with HFpEF underwent exercise testing with concomitant administration of sublingual nitroglycerin. Expectedly, the increase in PCWP during exercise was attenuated, but there was no change in peak VO_2_. The findings of this study suggested that decreasing PCWP in isolation may not be an effective strategy to improve exercise capacity in patients with HFpEF and brings into question whether the rise in PCWP itself is indeed the primary pathogenic factor leading to exercise intolerance. Subsequent work by the same group showed that while nitroglycerin administration was associated with decreased PCWP, there was an increase in ventilation-perfusion (V/Q) mismatch, perceived breathlessness, and evidence of worsened ventilatory inefficiency [142]. It should be noted that while a decrease in PCWP may directly lead to deleterious changes in pulmonary function, it may also be that such ventilatory impairments are related to nitrate use itself. By virtue of their mechanism of action as a venodilator at low doses, nitrates may also contribute to a reduction in preload reserve. Indeed, diminished preload reserve has also recently been implicated as a potential contributory mechanism in HFpEF exercise intolerance [143]. Certainly, further study is needed to understand if the conventional reasoning that elevated PCWP is the primary cause of exercise intolerance and is not, in fact, a secondary effect of the hemodynamic derangements that occur in HFpEF during exercise.

## HFpEF vs. HFrEF

While HFpEF is commonly described as a different disease process than the underlying cardiomyopathies that lead to HFrEF, they share many common mechanisms that contribute to exercise intolerance. These include reductions in stroke volume reserve, chronotropic incompetence, and impaired peripheral oxygen extraction [92]. Additionally, comorbidities such as iron deficiency and pulmonary vascular dysfunction are often present in both groups [144–146].

Though a significant overlap of pathophysiologic mechanisms exists between the two groups, the degree to which they contribute to exercise intolerance does appear to vary. For instance, it has been previously shown that peripheral oxygen extraction may be more impaired in those with HFpEF than HFrEF [92], which appears to be related to changes in previously discussed skeletal muscle and vascular function. In addition, left atrial function may also be disproportionately impacted in patients with HFpEF, as evidenced by more pronounced reductions in peak left atrial strain during exercise [147]. Conversely, diminutions in stroke volume reserve appear to be more prominent in patients with HFrEF [92]. Indeed, previous exercise testing has shown that patients with HFrEF may have modestly greater impairment in peak VO_2_ and lower pulse pressure at peak exercise compared to those with HFpEF, though other measures of exercise such as ventilatory anerobic threshold are similar [10]. Therefore, reductions in peak VO_2_ and narrower pulse pressure are likely related to LV systolic dysfunction with greater reductions in contractile reserve.

Still, the overlap between HFpEF and HFrEF is substantial, and the distinction between the two conditions is at times difficult to make, particularly at the margins of their defined ejection fractions (i.e., 40–60%). For example, a recent study performed a multimodal evaluation of patients with HFpEF across a range of ejection fractions [148]. They found that subjects with ejection fractions between 50 and 60% exhibited decreased LV contractility and ventriculo-arterial coupling with higher levels of fibrosis compared to those with EF > 60%, with the latter cohort demonstrating an excessive response to increased afterload and impaired preload reserve. Broadly, those subjects with ejection fractions at the lower range of HFpEF in some ways appear to have similar structural and functional changes to those seen in HFrEF. It might be that an obese patient with HFrEF (EF 38%) exhibits a much more similar exercise response to that of an obese patient with HFpEF (EF 50%) than either share with a lean patient with HFpEF (EF 70%). As our collective understanding of these disease processes to continues to improve, we may find that distinguishing between HFrEF and HFpEF as a means to diagnose and treat exercise intolerance is less than ideal.

## Conclusion

**Fig. 1:**
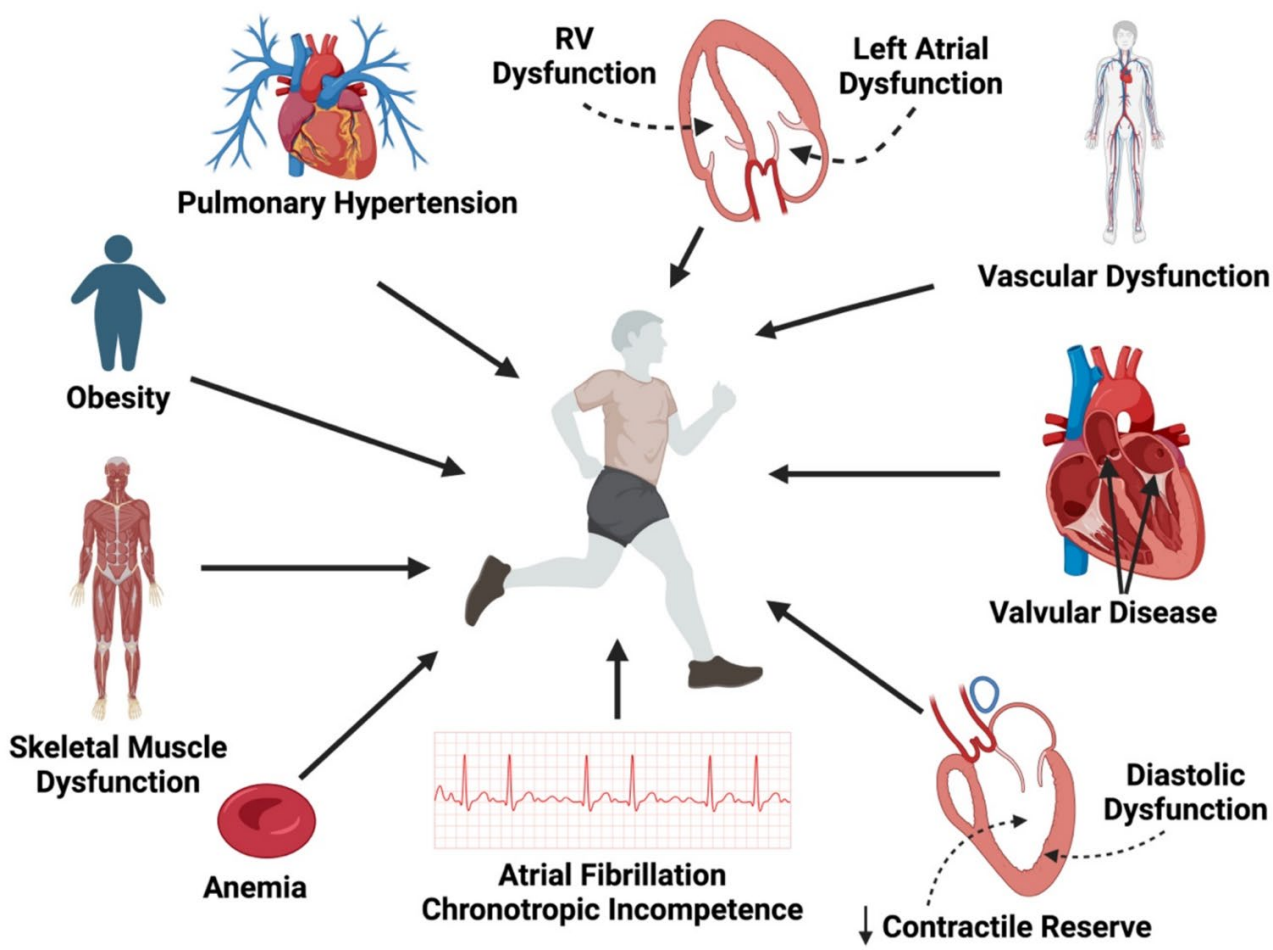
Mechanisms of exercise intolerance in heart failure with preserved ejection fraction (HFpEF). Contributory factors include diastolic dysfunction of the left ventricle, reduced contractile reserve, valvular disease, vascular dysfunction, left atrial dysfunction, right ventricular (RV) dysfunction, pulmonary hypertension, obesity, skeletal muscle dysfunction, anemia, atrial fibrillation, and chronotropic incompetence. This figure was created using BioRender.com

As described in this review, exercise intolerance in HFpEF involves more than isolated diastolic dysfunction of the left ventricle. A summary of contributory mechanisms can be seen in Fig. 1, and a summation of physiologic changes in those with HFpEF compared to normal subjects is shown in Table 1. Additional pathology of the left heart may include impaired LV contractile reserve, aortic and mitral valve disease, and left atrial dysfunction. The respiratory system, including the dysfunction of pulmonary vasculature and respiratory muscle mechanics, has also been implicated. Further, numerous other factors such as anemia, hormonal dysregulation, skeletal muscle dysfunction, and obesity are likely contributory. The degree to which each of these mechanisms contributes to exercise intolerance may vary considerably depending on an individual’s structural heart and vascular changes, along with one’s comorbidities. And despite a more holistic view of HFpEF, recent studies have even challenged our understanding of the basic principles of this disease process. As such, patients with HFpEF and exercise intolerance may be best treated with attention to each of these contributory factors.

**Table 1:** Summary of central and peripheral mechanisms of exercise response in HFpEF compared to normal subjects [32, 80, 125, 149–152]

	HFpEF	Normal
Left Ventricle	↑EDV, ↑↑↑EDP	↑EDV, ↑EDP
Left Atrium (LA)	↑Size, ↓Compliance, ↑↑Pulmonary venous pressure	↑Compliance, ↓Pulmonary venous pressure
Pulmonary Circulation	↑PVR, ↑↑↑mPAP	↑Pulmonary capillary recruitment, ↓PVR, ↑mPAP
Heart Rate (HR)	↑HR	↑↑HR
Vascular	↑Inflammation, ↓NO, ↑Stiffness, ↑Fibrosis	↑NO, ↑Vasodilation^3^
Lungs	↑↑Pulmonary pressure, ↑Permeability into alveolar space, ↓Surface area for gas exchange	↑Pulmonary capillary distension, ↑Surface area for gas exchange
Skeletal Muscle	↑↑↑Sympathetic drive, ↑Vasoconstriction, ↑Afterload, ↓Skeletal muscle perfusion	↑Sympathetic drive, ↑Skeletal muscle vasodilation, ↑Skeletal muscle perfusion
Neurohormonal	↑↑↑Sympathetic drive, ↑↑↑RAAS activation, ↑↑↑Vasoconstriction	↑Sympathetic drive, ↑RAAS activation, ↑Vasoconstriction
Anemia	↓Oxygen carrying capacity, ↓VO2	↑Oxygen carrying capacity, ↑VO2
Obesity	↑Ventricular interdependence, ↑↑mPAP, ↑PVR, ↑↑↑mPAP	↓PVR, ↑mPAP

Abbreviations: *EDV*, end-diastolic volume; *EDP*, end-diastolic pressure; *LA*, left atrium; *PVR*, pulmonary vascular resistance; *mPAP*, mean pulmonary arterial pressure; *NO*, nitric oxide; *N*, normal; *RAAS*, renin–angiotensin–aldosterone system; *VO2*, peak oxygen uptake

Lastly, in addressing exercise intolerance in patients with HFpEF, one must consider the goals of treatment. In patients with HFrEF, there exists clear treatment in strategies in the form of guideline-directed medical therapy (GDMT), which is sometimes erroneously referred to as “goal-directed medical therapy.” Like its counterpart, HFpEF imposes significant exercise impairment in many patients to the degree in which they are no longer able to maintain functional independence [13]. It is unlikely that many of those with HFpEF aspire to partake in high-intensity exercise but rather hope to perform the usual activities of daily living without significant compromise. Currently, the goal of functional independence may not be as readily and specifically captured by traditional means of assessment (i.e., cardiometabolic and hemodynamic testing, 6-min walk distance, and even quality of life questionnaires). Perhaps, a new model of assessment could lead to the development of true *goal-directed medical therapy* for HFpEF that is individualized for each patient. It will be crucial to keep this concept in mind as we work to better understand and treat HFpEF and its associated exercise intolerance.

## Data Availability

Data sharing is not applicable to this article as no new data were created or analyzed in this study.
